# Association of Temperament with Growth Performance in Nili Ravi Buffalo Heifers

**DOI:** 10.3390/ani15152255

**Published:** 2025-07-31

**Authors:** Salman Khalid Gorsi, Hamza Manzoor, Muhammad Qamer Shahid

**Affiliations:** 1 Department of Livestock Management, University of Veterinary and Animal Sciences, Lahore 54000, Pakistan; drskgorsi@gmail.com; 2 Department of Theriogenology, University of Veterinary and Animal Sciences, Lahore 54000, Pakistan; hamzamanzoor431@gmail.com

**Keywords:** dairy buffalo heifers, temperament, average daily gain, cortisol level

## Abstract

This study investigated the association between temperament and retrospective growth rates in 84 Nili Ravi buffalo heifers aged 18 to 24 months. Temperament was assessed using chute score and exit velocity, measured twice at a seven-day interval, and classified as calm (≤3) or nervous (>3). Retrospective average daily weight gain data were retrieved from farm records, and blood samples were collected to measure cortisol levels. ANOVA was used to analyze data, considering temperament, age group, season, and year of birth as fixed effects, with birth weight as a covariate. Results showed that 48 heifers were calm and 36 were nervous. Calm heifers exhibited significantly higher average daily gains than nervous heifers during the post-weaning period, with an increase of 240 g/day from 4 to 6 months and 190 g/day from 6 to 12 months (*p* < 0.001). However, this difference was not significant at 18–24 months (*p* = 0.144). Calm heifers have numerically lower cortisol levels (0.96 vs. 1.27 μg/dL; *p* = 0.11). These findings suggest that calmer heifers grow faster in early life, emphasizing the importance of temperament in breeding programs aimed at improving growth performance and welfare.

## Simple Summary

This study explored the relationship between temperament and growth performance in Nili Ravi buffalo heifers. A total of 84 heifers, aged 18 to 24 months, were assessed for temperament using chute score and exit velocity. Based on these measurements, heifers were classified as either calm or nervous. Retrospective growth data, including average daily gain, were obtained from farm records, and blood samples were analyzed for cortisol levels. Results showed that 48 heifers were classified as calm, while 36 were nervous. Calm heifers had significantly higher average daily gains than nervous heifers, particularly during early growth phases (4–6 months: +240 g/day; 6–12 months: +190 g/day). However, this advantage was not observed at 18–24 months. Additionally, calm heifers have numerically lower cortisol levels (0.96 vs. 1.27 μg/dL). These findings emphasize the importance of temperament in improving growth performance, highlighting its potential role in breeding and management strategies for dairy buffalo.

## 1. Introduction

Temperament refers to the behavioral responses of animals to stressful situations, such as handling and restraint, and is a key predictor of animal welfare and productivity [1]. It is typically consistent throughout an animal’s life [2] and is influenced by both genetic and environmental factors [3]. Temperament is a moderately heritable and repeatable trait, making it a suitable consideration in selective breeding programs [4]. Various approaches have been used to assess temperament, including observation of behaviors during restraint, such as kicking, vocalization, and escape attempts, and measuring escape velocity from chutes [5,6]. These assessments offer standardized, low-cost indicators that can be used in breeding goals to improve livestock productivity and well-being.

Research on cattle suggests that animals with calmer temperaments generally demonstrate improved feed efficiency and enhanced growth performance [7,8]. In Simmental and Nellore cattle, docile individuals outperformed excitable counterparts in growth and carcass quality [8,9]. Murrah buffaloes with calmer temperaments produced more milk and resumed ovarian activity sooner [10]. Excitable animals often exhibit elevated cortisol levels, which can disrupt hypothalamic-pituitary-adrenal (HPA) axis regulation, reduce dry matter intake, and ultimately limit weight gain [11,12]. These effects are reflected in feedlot and finishing systems where excitable animals often show reduced weight gain and carcass value [7]. Selecting for calm temperament supports improved growth, reproductive performance, meat tenderness, and overall farm profitability.

Although temperament-growth associations are well studied in Bos taurus and Bos indicus breeds, the evidence is lacking for dairy buffaloes, especially in the economically significant Nili Ravi breed. Early growth influences the age at first calving and subsequent lifetime milk production, underscoring the need to understand temperament’s role during the heifer stage. However, most buffalo studies focus on lactating animals, with little attention to growth trajectories during development [10].

The objective of this study was to examine the relationship between temperament—measured using chute scores and exit velocity—and retrospective growth performance in Nili Ravi buffalo heifers. We hypothesized that calmer heifers would show improved growth, possibly due to reduced physiological stress. Understanding this association may help inform management practices that promote both productivity and animal welfare in buffalo production systems.

## 2. Materials and Methods

### 2.1. Study Site, Animals and Management

This study was conducted at the Livestock Experimental Station, Haroonabad, Pakistan. A total of 84 healthy Nili Ravi buffalo heifers aged 12–24 months were selected from the station herd. Animals were reared under a loose housing system and managed according to standard farm protocols. All heifers were provided with seasonal green fodder and concentrates according to their age and nutritional requirements and had ad libitum access to fresh water.

### 2.2. Temperament Assessment

Temperament was assessed for each heifer using chute score and exit velocity. The observer received prior training in both assessment methods to ensure consistency and accuracy. Each animal underwent two complete temperament assessments—each consisting of both chute score and exit velocity—conducted one week apart during the morning hours (approximately 1000 h). All handling was performed by familiar stockpersons to minimize stress-induced behavioral variability. Data collection took place in March. For temperament classification, only the first assessment was used due to unforeseen weather conditions, specifically, rainfall on the day preceding the second assessment. Although the chute scores appeared unaffected, exit velocity values were noticeably lower, likely due to the wet and slippery ground surface. To avoid potential misclassification of temperament, only the first assessment—conducted under consistent and dry conditions—was considered for analysis.

#### 2.2.1. Chute Score Assessment

Chute score was evaluated based on each animal’s behavior while restrained in a chute, without the application of any external stimuli. Animals were restrained by trained farm attendants responsible for routine husbandry practices. The dimensions of the experimental chute were recorded and measured approximately 2.4 m in length, 0.9 m in width, and 1.5 m in height. Each animal was restrained in the chute for approximately 3 min during the chute score evaluation. A camera was positioned to record each animal’s behavior during restraint. One trained observer reviewed the video footage and assigned a chute score based on the animal’s activity within the chute. Scoring was conducted using a standardized chart adapted from Parham and Lewis [6] (Table 1).

#### 2.2.2. Exit Velocity Score

Exit velocity was measured following the method described by Cooke et al. [13]. Immediately after chute scoring, the time each heifer took to cover a distance of 1.9 m upon exiting the chute was recorded using a stopwatch. Exit velocity was then calculated using the following formula:

Exit Velocity (m/s) = Distance Covered (m)/Time Taken (s)

where the distance was fixed at 1.9 m.

Based on the calculated exit velocity, animals were equally divided into quintiles. Each heifer was then assigned an exit velocity score ranging from 1 to 5, with a score of 1 representing the slowest animals and a score of 5 representing the fastest. This scoring method was adapted from Cooke et al. [13].

#### 2.2.3. Temperament Score

The final temperament score was calculated as the average of the chute score and the exit velocity score, using the following formula:

Temperament Score = (Chute Score + Exit Velocity Score)/2

Based on the calculated temperament scores, animals were classified into two categories: those with a temperament score ≤ 3 were considered calm, while those with a score > 3 were classified as nervous. In some studies, this classification has also been described using the terms adequate (or calm) and excitable (or nervous), depending on the specific threshold criteria applied for grouping.

### 2.3. Blood Sampling for Cortisol Level

Blood samples were collected once from the jugular vein of each experimental animal immediately following temperament scoring. Samples were drawn into sterile vacutainer tubes containing anticoagulant. Plasma was separated by centrifugation, and all samples were handled under a strict cold chain to preserve hormone integrity throughout the collection and processing procedures. Cortisol concentrations were determined using commercially available colorimetric assay kits, and absorbance was measured using a spectrophotometer (Epoch2, BioTek Instruments, Winooski, VT, USA).

### 2.4. Heifer Performance Variables

The following variables were recorded: date of birth, birth weight, weaning weight, weaning age, body weight at regular six-month intervals, and the season and year of birth. To minimize potential confounding effects on growth performance, date of birth was used to classify each animal into corresponding birth season and birth year categories.

The growth data used in this study were obtained from official records maintained at the Livestock Experiment Station, where a standardized protocol is routinely followed for body weight recording. Specifically, weights such as birth weight, weaning weight, and monthly body weights are measured and documented during the first week of each month for all animals in the herd. All heifers under two years of age (*n* = 84) that were present at the farm and had complete historical weight records in the farm register were selected for this study. The records were carefully examined to ensure they were complete and consistent before being used in the analysis. Since no data were missing, there was no need for imputation.

### 2.5. Statistical Analysis

All the statistical analyses were performed using SAS software (SAS 9.4M8, SAS Institute Inc., Cary, NC, USA). The variables related to calm and nervous heifers were first descriptively presented as means and standard deviations. Weighted Kappa statistics was applied to determine the validity of the scoring systems. To assess the association of temperament and growth performance, the collected data were subjected to analysis of variance using the MIXED procedure of SAS. The temperament trait, the age group of heifers, the season of birth and the year of birth were considered as fixed effects, and birth weight as a covariate. The significance was set at *p* ≤ 0.05.

## 3. Results

### 3.1. Chute Score

**Figure 1:**
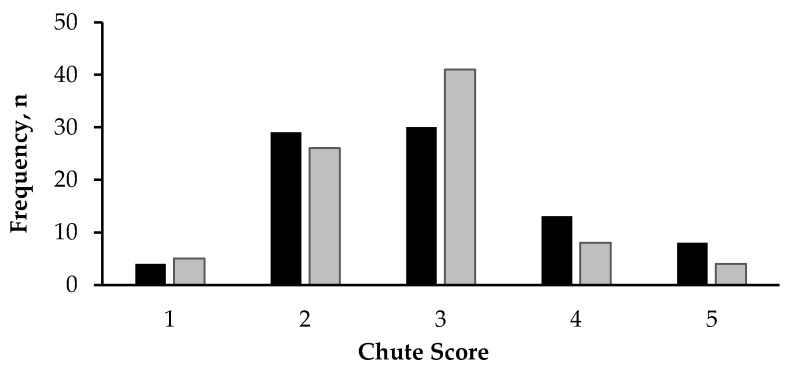
Frequency distribution of chute scores across two assessments (Assessment 1: black bars; Assessment 2: grey bars; *n* = 84). The two assessments were conducted one week apart.

Chute score was a more subjective component of the temperament assessment. The frequency distribution of chute scores assigned to the heifers is shown in Figure 1. The most commonly observed chute score was 3, followed by scores 2, 4, 5, and 1. Overall, the majority of heifers received chute scores of 2 or 3, indicating a moderate behavioral response during restraint.

The results of intra-observer reliability for chute scores are presented in Table 2. In the first assessment, the percentage of exact agreement (tolerance level 0) was 85%, while agreement within one score (tolerance level 1) reached 98%. In the second assessment, the percentage of exact agreement was 84%, and agreement within one score reached 100%, indicating perfect consistency at that tolerance level.

### 3.2. Descriptive Traits of Calm and Nervous Heifers

Based on chute score and exit velocity score, heifers were classified as calm or nervous. The descriptive traits associated with each temperament group are presented in Table 3. Calm heifers constituted 57% (*n* = 48) of the experimental population, while the remaining 43% (*n* = 36) were classified as nervous. Calm heifers had a mean chute score of 2.3, an average exit velocity of 1.2 m/s, and a mean exit score of 2.1. In contrast, nervous heifers exhibited a mean chute score of 4.0, an average exit velocity of 2.4 m/s, and a mean exit score of 4.5.

### 3.3. Association of Temperament with Growth Performance

The association between heifer growth and temperament is presented in Table 4. Retrospective data showed that both calm and nervous heifers had similar birth weights (37.7 ± 0.23 kg). However, by weaning, calm heifers tended to weigh approximately 5 kg more than their nervous counterparts. The difference in growth became more pronounced with age, with calm heifers exhibiting significantly higher body weights at 6, 12, and 18 months compared to nervous heifers. At six months of age, calm heifers had an average body weight that was 21 kg higher than that of nervous heifers. Similarly, at one year of age, calm heifers were, on average, 52 kg heavier than their nervous counterparts. At 18 months, the average weight difference remained substantial, with calm heifers weighing 43 kg more than nervous heifers.

Temperament significantly influenced average daily gain (ADG) during specific growth phases. Between 4 and 6 months of age, calm heifers outperformed nervous heifers by 240 g/day (*p* < 0.05). This advantage persisted, though slightly diminished, during the 6–12-month period, with calm heifers maintaining an 180 g/day higher ADG (*p* < 0.05). However, no significant temperament-related differences in ADG were detected at weaning, or between 12 and 24 months of age (*p* > 0.05).

**Figure 2:**
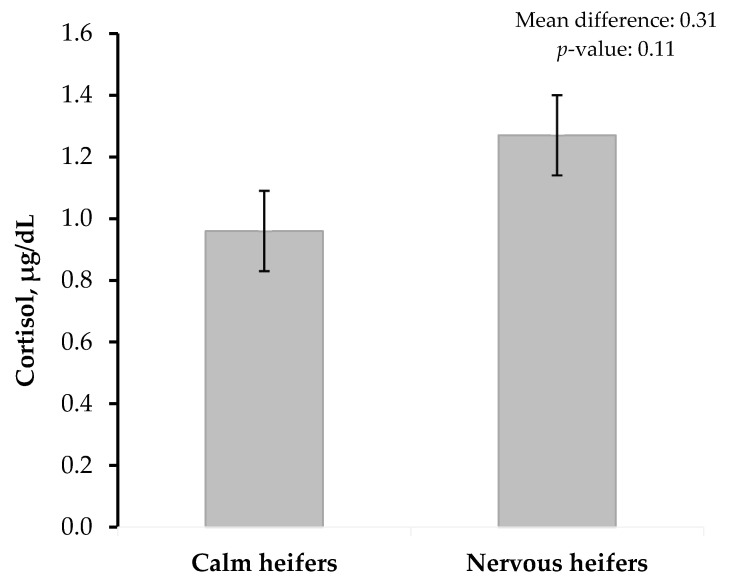
Cortisol levels in Nili Ravi buffalo heifers classified as calm or nervous based on temperament assessment (*n* = 84).

The results indicated that cortisol levels were numerically higher in nervous heifers compared to calm ones, although the difference was not statistically significant (*p* = 0.110; Figure 2). Calm heifers had a mean cortisol concentration of 0.96 μg/dL, while nervous heifers exhibited a higher mean level of 1.27 μg/dL.

## 4. Discussion

As limited literature is available on temperament characteristics in Nili Ravi buffaloes, direct comparisons with other studies are challenging. However, our findings are consistent with those reported by Francisco et al. [14], who classified 65% of Nelore growing steers as adequately tempered, while the remaining 35% were categorized as excitable. It is important to note that several factors, such as the number of animals included in the study, the production system, and the temperament evaluation criteria, can significantly influence the outcomes of temperament assessments.

Studies in cattle have demonstrated that animal temperament can be influenced by repeated human handling over time [15]. In contrast, research on Indian cattle (Bos indicus) and their crossbreds has shown that animals tend to exhibit consistent temperament traits over time, suggesting a degree of repeatability in behavioral responses [12]. In management systems such as loose housing or free-stall operations, where human–animal interactions are limited, animals are more likely to exhibit consistent behavioral responses over time. This consistency may be even more pronounced in larger herds, where individual handling is minimal, although this remains speculative and warrants further investigation.

In our study, calm heifers exhibited higher average body weight between 6 and 18 months of age. Similarly, calm-tempered heifers demonstrated greater average daily gain (ADG) during the 4–12-month period. This association between nervous temperament and reduced growth performance has been well-documented in previous studies [1,7,8]. Excitable animals are known to have elevated baseline energy requirements compared to calmer counterparts [16], even in the absence of acute stressors [12,17]. Moreover, the behavioral characteristics of nervous animals, such as increased physical activity and heightened arousal, may contribute to reduced growth efficiency [18]. These behaviors also decrease time allocated to rumination and rest, which are essential for growth and metabolic recovery. Furthermore, a negative association between high flight speed and dry matter intake has been reported, suggesting that nervous or excitable animals may have compromised feed intake behavior [7], which could contribute to reduced growth rates.

The significant differences in growth rate observed during the post-weaning period, particularly between 6 and 18 months of age, may suggest that temperament plays a more critical role once heifers become nutritionally independent. During the pre-weaning phase, when milk was fed by humans, nervous heifers were likely less affected, as their feeding was assisted. However, following weaning, when animals had to rely on self-feeding, nervous heifers may have exhibited altered feeding behavior, which negatively impacted their growth performance. This disadvantage became evident immediately after weaning and persisted through the 6–12-month period, with calm heifers consistently exhibiting higher growth rates. Although growth differences diminished by 18–24 months, the initial advantage held by calm animals underscores the potential long-term impact of early-life behavioral traits.

The overall low growth performance observed in the study also indicates that Nili Ravi buffalo heifers face considerable stress in the post-weaning phase. Nervous-tempered heifers, in particular, may allocate more energy toward coping with stress and maintaining a heightened state of arousal, rather than toward productive functions such as growth. This inefficiency likely contributes to their reduced feed utilization and overall poorer performance compared to calm counterparts.

In our study, excitable animals tended to exhibit numerically higher cortisol levels compared to calm heifers. Cortisol is widely recognized as a key physiological indicator of stress, and elevated levels are typically associated with exposure to stressors or anxiety [19]. In agreement with current findings, previous research has reported that excitable cattle often display higher blood cortisol concentrations than calmer individuals [20]. However, given the low statistical significance (*p* = 0.11), these results should be interpreted with caution. Further research involving a larger sample size is necessary to confirm and strengthen this association. Furthermore, alternative approaches to temperament classification, such as treating it as a continuous variable, should also be explored in future studies.

However, findings in the literature are not entirely consistent. For instance, Francisco et al. [14] reported no significant interaction between cortisol levels and temperament in their study. Instead, they observed that cortisol concentrations were more strongly influenced by dietary interventions than by behavioral classification. These contrasting results suggest that the relationship between temperament and cortisol may be context-dependent, influenced by environmental, nutritional, and management factors. Additionally, as cortisol levels were recorded post-handling, future studies should incorporate baseline (pre-handling) cortisol measurements to more accurately assess the physiological stress profile of animals in relation to temperament.

## 5. Conclusions

In conclusion, this study demonstrates a clear association between temperament and growth performance in Nili Ravi buffalo heifers. Calm heifers (chute score ≤ 3) exhibited significantly higher average daily gains than nervous heifers during, particularly from 4 to 12 months of age, indicating that temperament influences growth most strongly in the early developmental stages. The numerically lower cortisol levels observed in calm heifers suggest a potential physiological link between reduced stress and improved growth efficiency. However, further investigations with a larger sample size are required to validate and strengthen this association.

These findings underscore the value of incorporating temperament assessment into buffalo management and breeding strategies. Selecting for calmer temperament may not only enhance productivity through improved growth performance but also contribute to better animal welfare by reducing stress. Future research should focus on the genetic basis of temperament traits and their long-term effects on reproductive performance, metabolic efficiency, and stress resilience in buffalo production systems.

## Figures and Tables

**Table 1:** Chute score criteria based on observed behavior during restraint.

Score	Classification	Observed Behavior
1	Docile	Stands very calmly; slow movement; undisturbed; no tail flicking most of the time; normal ear movement.
2	Slightly Restless	Occasional tail flicking; head movement; slight movement of any leg.
3	Restless	Occasional tail and head movement; head movement with pushing and pulling, with or without leg movement.
4	Nervous	Continuous head movement with pushing and pulling (with or without leg movement); occasional eye white; frequent tail flicking; impatient.
5	Flighty or Wild	Frightened; continuous tail flicking; eye white visible; salivation or frothing from the mouth; jumpy and difficult to control.

**Table 2:** Weighted kappa coefficients for intra-assessor agreement on chute scores in Nili Ravi buffalo heifers (*n* = 84), including exact agreement and agreement within ±1 score.

Scoring	No. of Scoring Points	Chute Scoring Range ^1^	Level of Agreement	Estimate	SE	95% Confidence Limit
Scoring I						
	5	1 to 5	Exact	0.85	0.04	0.79–0.93
	5	1 to 5	±1	0.98	0.02	0.93–1.0
Scoring II						
	5	1 to 5	Exact	0.84	0.04	0.76–0.92
	5	1 to 5	±1	1.0		1.00–1.00

^1^ Chute scoring was conducted as described earlier, using a scale from 1 (docile) to 5 (flighty or wild), with one-point increments.

**Table 3:** Descriptive statistics of temperament-related variables—chute score, exit velocity, and exit score—in calm and nervous Nili Ravi buffalo heifers (*n* = 84).

Items	Mean	SD	Minimum	Maximum
Calm Heifers ^1^, *n* = 48				
Chute score,	2.3	0.58	1.0	3.0
Exit velocity, m/s	1.2	0.29	0.7	2.3
Exit velocity score	2.1	0.86	1	4
Nervous Heifers, *n* = 36				
Chute score,	4.0	0.55	3.5	5.0
Exit velocity, m/s	2.4	0.54	1.25	4.0
Exit velocity score	4.5	0.57	3	5

^1^ Buffalo heifers with a temperament score ≤ 3 were classified as calm, whereas those with a score > 3 were considered nervous.

**Table 4:** Retrospective growth performance of Nili Ravi buffalo heifers classified by temperament (calm vs. nervous) (*n* = 84).

Variables	Temperament Classes		SE	Mean Difference	*p* Values
	Calm Heifers	Nervous Heifers			
Average weight, kg					
At birth	37.6	37.9	0.23	−0.32	0.207
At weaning	105.5	100.6	2.3	4.96	0.074
At 6 months	130.1	109.3	3.7	20.79	<0.0001
At 12 months	204.0	151.6	7.0	52.45	<0.0001
At 18 months	256.8	213.3	9.5	43.50	<0.0001
At 24 months	294.6	279.3	10.3	15.28	0.147
Average Daily Gain, kg/d					
Birth to weaning	0.55	0.51	0.02	0.04	0.158
4–6 months	0.38	0.14	0.04	0.24	<0.0001
6–12	0.42	0.23	0.03	0.18	<0.0001
12–18	0.29	0.33	0.02	−0.04	0.1449
18–24	0.22	0.25	0.02	−0.03	0.0663

## Data Availability

The data presented in this study are available on a reasonable request from the corresponding author.
